# Vestibular Deficit in Patients with Waardenburg Syndrome

**DOI:** 10.3390/biomedicines13082021

**Published:** 2025-08-19

**Authors:** Mathilde Benifla, Margaux Serey-Gaut, Emilie Bois, Salma Jbyeh, Natacha Teissier, Monique Elmaleh-Bergès, Laurence Jonard, Véronique Pingault, Natalie Loundon, Kahina Belhous, Sandrine Marlin, Audrey Maudoux

**Affiliations:** 1 Department of Otolaryngology, Robert Debré Hospital, AP-HP, Faculty of Medicine, Paris University, F-75019 Paris, France; 2 Department of Genetics, Necker Hospital, CRMR Surdités Génétiques, Service Médecine Génomique des Maladies Rares, UF Développement et Morphogenèse, AP-HP, Faculty of Medicine, Paris University, F-75015 Paris, France; 3 Centre de Recherche en Audiologie, Necker Hospital, AP-HP, Faculty of Medicine, Paris University, F-75015 Paris, France; 4 Department of Pediatric Radiology, Robert Debré Hospital, AP-HP, Faculty of Medicine, Paris University, F-75019 Paris, France; 5 Department of Otolaryngology, Necker Hospital, AP-HP, Faculty of Medicine, Paris University, F-75015 Paris, France; 6 Department of Pediatric Radiology, Necker Hospital, AP-HP, Faculty of Medicine, Paris University, F-75015 Paris, France; 7 Unité INSERM UMR1163, Institut Imagine, Génétique des Maladies Ophtalmologiques Auditives et Mitochondriales Rares, F-75015 Paris, France; 8 Institut Pasteur, Institut de l’Audition, Unit Progressive Sensory Disorders Pathophysiology and Therapy, Université Paris Cité, INSERM A006, F-75012 Paris, France

**Keywords:** vestibular function, balance, Waardenburg syndrome, inner ear malformations, hearing loss

## Abstract

**Background/Objectives**: Waardenburg syndrome (WS) is a genetic disorder characterized by sensorineural hearing loss (SNHL) and pigmentation anomalies. While hearing impairment is a well-established feature of WS, vestibular dysfunction is also reported. This study aimed to investigate vestibular deficits in pediatric WS patients with SNHL, correlating these findings with molecular, audiometric, and radiological data to establish distinct phenotypic profiles for each WS subtype and associated pathogenic variants. **Methods**: This retrospective study included children with a genetically confirmed diagnosis of WS who underwent vestibular, auditory, and inner ear radiological assessments as part of their routine medical care between July 2000 and May 2022. Data were collected from medical records, including medical history, clinical findings, and assessment results. **Results**: Vestibular dysfunction was found to be highly prevalent, affecting 64% of the cohort, often impacting the canal sensory organ (89%) and occasionally the otolithic function (33%). Patients with *SOX10* pathogenic variations exhibited a markedly higher risk of vestibular dysfunction, highlighting the unique role of *SOX10* in inner ear development. Notably, inner ear malformations were identified in all *SOX10*-mutated subjects, whereas such anomalies were rare among individuals with other WS gene variants, occurring in only two additional cases with minor malformations. **Conclusions**: This study reveals a significant prevalence of vestibular deficits in pediatric WS patients with SNHL, emphasizing the need for routine vestibular assessments. The higher prevalence and severity of vestibular impairments in *SOX10*-mutated patients underscore the importance of molecular analysis in clinical diagnosis and management.

## 1. Introduction

Waardenburg syndrome (WS) is a genetic disorder characterized by sensorineural hearing loss (SNHL) and pigmentation anomalies, including depigmented patches of skin and hair, as well as vivid blue eyes or iris heterochromia. First described in 1951 by the Dutch ophthalmologist and geneticist Petrus Johannes Waardenburg [1], the syndrome has an estimated incidence of approximately 1 in 40,000 births and accounts for 1–3% of congenital deafness cases [2]. The clinical features of WS exhibit significant inter- and intra-familial variability in both the degree and pattern of expression [3]. Four types of WS are clinically defined, based on features due to defects in structures mostly arising from neural crest derivatives: WS type 1 is further characterized by dystopia canthorum (OMIM #193500), WS type 3, also known as Klein–Waardenburg syndrome, by musculoskeletal abnormalities of the limbs (OMIM #148820), WS type 4, or Shah-Waardenburg syndrome, by Hirschsprung disease (OMIM #277580), whereas WS type 2 has no further specific significant features (OMIM #193510).

Six genes are involved in this syndrome [3]: *PAX3* (paired box 3 transcription factor) in WS type 1 and type 3; *MITF* (microphthalmia-associated transcription factor) in WS type 2; *EDN3* (endothelin 3) in WS type 4; *EDNRB* (endothelin receptor type B) in WS type 4 and type 2; *SOX10* (Sry bOX10 transcription factor) in WS type 4, type 2, and in WS with neurological features (called PCWH for Peripheral demyelinating neuropathy, Central dysmyelination, Waardenburg syndrome, and Hirschsprung disease OMIM #609136); and *KITLG* (KIT Ligand) in WS type 2.

While hearing impairment is a well-established feature of WS, vestibular dysfunction is less frequently reported. However, given the anatomical continuity and the structural and developmental similarities between the cochlea and the vestibular labyrinth, similar developmental anomalies and pathogenic mechanisms may affect both structures [4]. Moreover, imaging studies have previously documented labyrinthic abnormalities in WS, mostly due to *SOX10* pathogenic heterozygous variations [2,5], yet their correlation with vestibular dysfunction remains unexplored.

The objective of this study was to investigate vestibular deficit in WS patients with SNHL and to link these deficits to molecular, audiometric, and radiological findings, aiming to establish distinct phenotypic profiles for each WS subtype and associated pathogenic variants. Since vestibular deficits can significantly impact early motor and cognitive development in children [6], evaluating the prevalence and importance of vestibular assessment in this population is crucial.

## 2. Materials and Methods

This retrospective study included 34 children with a genetically confirmed diagnosis of WS (either by direct confirmation of a pathogenic variant through molecular genetic testing (n = 30), or, for a minority, through fulfillment of clinical diagnostic criteria combined with genetic confirmation of a pathogenic variant in a first-degree relative (n = 4)) who underwent vestibular, auditory, and inner ear radiological assessments as part of their routine medical care in the context of SNHL between July 2000 and May 2022 in two expert pediatric centers. Data were collected from medical records and included information on medical history, clinical findings related to WS features, and results of vestibular, auditory, and imaging assessments. Several children evaluated in the present study were also part of an earlier publication focusing on inner ear abnormalities in WS [2]. This study was conducted in accordance with the ethical guidelines of our institutional review board for retrospective observational studies (Comité d’Evaluation de l’Ethique des Projets de Recherche de Robert Debré, Nbr 2022-600). Statistical analyses were conducted using GraphPad Prism 10 (GraphPad Software, Boston, MA, USA, www.graphpad.com). Group comparisons were performed using the non-parametric Fisher’s exact test, and associations with walking age were assessed using multiple linear regression.

### 2.1. Genetic Assessment—Molecular Analysis

Written informed consent for genetic analysis was obtained from participants or their parents, for genetic testing in diagnosis settings. Over the years, patients were tested by Sanger Sequencing [7,8,9,10], Next-Generation Sequencing a panel of WS [11], Genome sequencing in trio [12], and for copy number variants by MLPA [13] or QM-PSF [14].

### 2.2. Vestibular Assessment

The vestibular system responds to sensory inputs across a range of frequencies, from low frequencies of approximately 0.05 Hz (e.g., postural sway) to high frequencies around 5 Hz during rapid head movements. Vestibular evaluation was conducted to systematically achieve the most comprehensive possible assessment of both canal and otolith function, incorporating neurological clinical evaluation and instrumental vestibular testing. Over the course of the study period, the vestibular assessment protocols aimed to be as consistent as possible, despite changes and improvements in diagnostic equipment (e.g., the introduction of vHIT and otolithic evoked potentials). Vestibular function was tested at various frequencies: (i) bicaloric test (30 and 44 °C) to assess canal function at low frequencies, (ii) rotatory chair to evaluate vestibulo-ocular reflex (VOR) at middle frequencies (MegaTorque Difra-Neurosoft^®^ system, Bruxelles, Belgium) and, (iii) video Head Impulse Test (vHIT) to evaluate the function of the six semicircular canals (SCC) at high frequencies (Synapsys system, Inventis^®^, Padova, Italy), or HIT in older cases. Otolithic function was assessed by using cervical vestibular evoked myogenic potential (c-VEMP) with short tone bursts (750 Hz, 4.1/s and 6 ms duration) delivered by air and/or bone conduction with control of the electromyogram level for each stimulation (Difra-Neurosoft^®^ system, Bruxelles, Belgium).

For the bithermal caloric test, the Jongkees formula was applied [15]. Values for relative valence and directional preponderance for children were considered normal when <15%. The bicaloric test responses could be either normal, partially normal (unilateral hyporeflexia or areflexia, bilateral hyporeflexia), or totally absent (bilateral areflexia).

For the rotary chair test, since VOR gain values may vary with age [16], gain values were considered normal if the interaural gain asymmetry was less than 30%.

For the vHIT, the presence of overt and covert saccades and the gain of each SCC were reported. The vHIT responses were classified as being normal (bilateral gains > 80% for lateral SCC and >70% for anterior and posterior SCC), partially normal (one or more gains between 20% and 80/70%), or totally absent (all 6 gains under 20%). In case only HIT was performed, it was classified as being normal (no catch-up saccades), partially normal (catch-up saccade in some SCC directions), or totally absent (catch up saccades in all 6 directions).

The cVEMP responses were interpreted in terms of P13 and N23 waves. Their latencies (ms), thresholds (dB), and amplitude (μV) at 100 dB were analyzed. The ratio between right and left amplitude related to the intensity of the muscular contraction was calculated. The cVEMP results could be either normal (presence of bilateral P13 and N23 waves with a symmetric amplitude at 100 dB: ratio < 40%), partially normal (unilateral hyporeflexia: unilateral positive responses only at thresholds greater than 100 dB and normal for the contralateral ear or a ratio > 40% at 100 dB or bilateral hyporeflexia: bilateral positive responses only at thresholds greater than 100 dB), or totally absent (absence of P13 and N23 waves at 110 dB).

Moreover, the age of acquisition of the posturomotor control milestones (head holding, sitting without support, standing with support, and independent walking) were carefully recorded for each child from their pediatric medical records or parental remembrance.

### 2.3. Hearing Assessment

The hearing assessment protocol was adapted to the child’s age. In newborns, auditory brainstem response (ABR) and/or auditory steady-state response (ASSR) testing was performed to determine the hearing threshold for each ear. For older children, hearing evaluation included behavioral audiometry combined with transient evoked otoacoustic emissions (TEOAE) or conventional pure-tone audiometry.

To standardize data and simplify the interpretation of hearing profiles, thresholds were categorized according to the International Bureau of Audiophonology (BIAP) classification as follows: normal hearing (≤20 dB), mild SNHL (21–40 dB), moderate SNHL (41–70 dB), severe SNHL (71–90 dB), and profound SNHL (>90 dB).

### 2.4. Imaging Assessment

All temporal bone CT scans were obtained on multisection CT scanners, with high-resolution helical acquisitions of ≤1 mm thick sections. Multiplanar reconstruction in the plane of each SCC was obtained. All MR imaging scans included at least an axial 3D high-resolution T2 TSE submillimeter sequence of inner ear structures and a whole-brain study with T2-weighted sequences (FLAIR and/or T2 TSE) in ≥1 plane (coronal and/or axial).

For each patient, the morphology of the inner ear was systematically evaluated to identify cochlear, vestibular, and/or semi-circular canal malformations. For each patient, visual analysis of the following inner ear structures was performed by experienced radiologists specialized in ENT and pediatric imaging (M.E.-B. and K.B.): cochlea (shape, number of turns, modiolus), vestibule (shape, size), SCCs (each was noted as absent or present, and when present, any anomaly in the shape and/or size was also described), and vestibular aqueduct/endolymphatic sac (size).

## 3. Results

### 3.1. Population Characteristics

Between July 2000 and May 2022, 34 patients (24 boys and 10 girls) with genetically confirmed WS and vestibular, hearing, and imaging assessment were included in this study (Table 1). The median age at inclusion, defined as the date of vestibular assessment, was 1.9 years old. Among the included patients, 11 have WS type 1 with *PAX3* pathogenic variations, 18 have WS type 2 with *MITF* (n = 4), *SOX10* (n = 13) or *EDNRB* (n = 1) pathogenic variations, and 5 have WS type 4 with *SOX10* (n = 4) or *EDNRB* (n = 1) pathogenic variations. No patients with WS type 3 were included (Table 2).

### 3.2. Vestibular Function

Due to the retrospective nature of the study, a complete and exhaustive vestibular evaluation was not obtained for all subjects. Nevertheless, comprehensive vestibular evaluation, incorporating at least one canal and one otolithic assessments, was conducted in 82% of our subjects (n = 28). In the remaining 18% of subjects, assessment was limited to canal function only (a detailed overview of the specific vestibular tests performed for each subject is provided in Table 1). Our initial analysis focused on subjects who had undergone at least one assessment of both canal and otolithic function. This approach ensured that the general prevalence estimates were robust and not overly influenced by incomplete assessments. In the second part, where canal and otolithic functions were analyzed separately, we also included subjects who had only undergone assessment of canal function.

**Figure 1:**
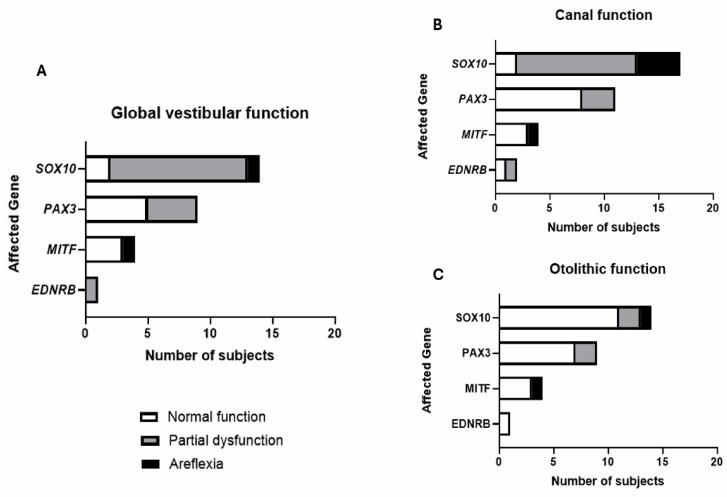
Distribution of normal, partial, and areflexic vestibular function by causative gene: (**A**) among subjects who underwent both canal and otolithic evaluations; (**B**) canal function and (**C**) otolithic function are presented separately for the entire cohort.

Looking at the 28 subjects with both canal and otolithic evaluation, vestibular function was found to be normal in 36% (n = 10) of the subjects, partially dysfunctional in 57% (n = 16), and completely absent (areflexia) in 7% (n = 2). Canal dysfunction (n = 16) was more frequent than otolithic dysfunction (n = 6). Previous studies have indicated that disease-causing genes are more predictive of the auditory phenotype than WS clinical subtypes (5). Consequently, to examine the impact of genotype on vestibular impairment, the presence and severity of vestibular dysfunction were categorized, for each of the 28 subjects with at least one canal and one otolithic assessment, according to the involved gene (Figure 1A). For patients with *PAX3* pathogenic variations, global vestibular function was normal in 56% (5/9) and partially dysfunctional in 44% (4/9). In patients with *MITF* pathogenic variations, vestibular function was normal in all but one, who exhibited total vestibular loss (areflexia). For patients with *EDNRB* pathogenic variations, one patient showed a slight unilateral canal dysfunction. Notably, for patients with *SOX10* pathogenic variations, global vestibular function was normal in 14% (2/14), partially dysfunctional in 79% (11/14), and completely absent in 7% (1/14). When analyzing the effects of the mutation on canal and otolithic function separately, and therefore including the six remaining patients with isolated canal function examinations, the impact of the *SOX10* pathogenic variations becomes even more pronounced (Figure 1B). Remarkably, 88% of patients with *SOX10* pathogenic variations exhibited abnormal canal function, with 24% having complete canal areflexia.

**Figure 2:**
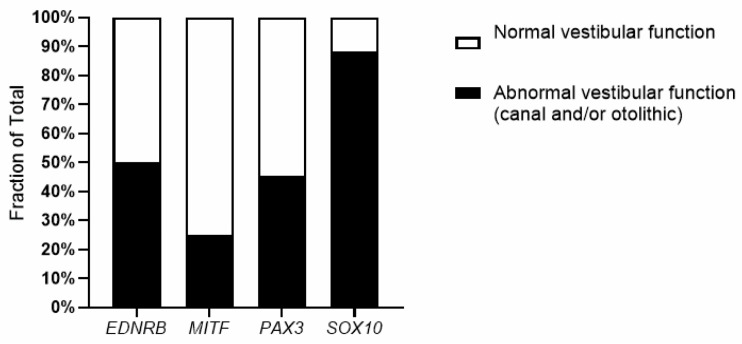
Vestibular function in patients according to the different WS-associated genes.

When comparing the prevalence of vestibular deficits—whether affecting the canals, the otolithic organs, or both—across the different genetic groups, we obtained a significant difference (*p* < 0.05 for group comparison, significant pairwise comparisons: *p* (*PAX3* vs. *SOX10*) < 0.05 and *p* (*MITF* vs. *SOX10*) < 0.05; non parametric Fisher Exact test) (Figure 2). These preliminary observations suggest potential variability in vestibular function based on specific genetic mutations and underscore the importance of genetic testing in predicting vestibular outcomes in patients with WS. Indeed, vestibular function is known to significantly influence a child’s motor and cognitive development. Moreover, severe vestibular deficits can lead to oscillopsia, causing visual impairments during movement, which in a population with HL may further compromise visual compensation for auditory deficits.

In our study cohort, we assessed the age at which children began walking independently, a key indicator of motor development. Multiple factors, including vestibular dysfunction and neurological involvement, can affect the age at which children acquire independent walking. Since neurological symptoms are mainly found in WS patients with *SOX10* pathogenic variants, we performed a multiple linear regression analysis to assess the association between age at independent walking, vestibular dysfunction, and the presence of a *SOX10* pathogenic variant. Of the two factors studied, only *SOX10* pathogenic variation was significantly associated with delayed walking age (β = 5.4 months; 95%CI [2.6–8.2]; *p* = 0.0005, R^2^ = 0.45). This association remained significant both when including and when excluding the two subjects with SOX10 variants who exhibited notably delayed walking age compared to the rest of the cohort.

### 3.3. Audiological Findings

Bilateral severe to profound SNHL was observed in all but three patients, with all cases being congenital in origin. Of the three patients with unilateral HL, two with *PAX3* pathogenic variations exhibited profound HL on the affected side, while one with a *SOX10* pathogenic variations had mild HL. For these three patients, the vestibular function was completely normal, except for a slight canal dysfunction in the normal hearing ear in one of the patients with unilateral HL and *PAX3* pathogenic variations.

### 3.4. Imaging Findings

Inner ear malformation was present in 55% of our study population. Among those with inner ear malformation, 79% had combined cochlear and vestibular/SCC malformations, while 21% had isolated vestibular/SCC malformations. Notably, all but two of these patients exhibited a *SOX10* pathogenic variation; the exception was two patients with a *PAX3* pathogenic variation, who presented with moderate inner ear malformation: an enlarged vestibular aqueduct for one, and a double ampulla-like appearance of the SCC for the other. When present, the most common anomaly involved the semi-circular canals (95%), while the vestibule/otolithic area was less frequently affected (68%). Interestingly, the presence of cochleovestibular malformation significantly increased the risk of abnormal vestibular function. Specifically, 84% of subjects with vestibular malformations exhibited abnormal vestibular function, compared to 40% of those without inner ear malformations (Fisher’s exact test *p* = 0.012).

## 4. Discussion

In this comprehensive study, we have meticulously analyzed the phenotypic profiles of patients diagnosed with WS, with particular emphasis on vestibular function. Previous investigations have predominantly focused on HL [5] and temporal bone abnormalities [2,5,17,18], but our findings highlight the broader histopathological implication of WS and associated mutations, particularly in the vestibular domain of patients with SNHL. This distinction is critical for clinicians and researchers aiming to better understand and manage the multifaceted symptoms of WS.

Vestibular function in WS has been scarcely observed and reported, with only four articles focusing on the vestibular function of WS patients. These studies, although often limited in their vestibular evaluations (otolithic function was poorly reported) and not conducted in conjunction with molecular analysis, offer some insights into vestibular dysfunction. Marcus et al. studied 22 subjects from a single family affected by WS and reported vestibular abnormalities in all but one subject, suggesting that vestibular pathologic changes constituted the most prominent feature in this group [19]. Conversely, Hageman et al. examined 34 individuals from five families with WS (31 with WS type 1 and 3 with WS type 2) and found caloric abnormalities in five WS type 1 subjects [20]. Hageman concluded that the incidence of vestibular dysfunction in WS patients was no greater than in individuals with other types of hereditary deafness, thus not considering vestibular pathologic changes as an essential symptom complex in WS. More recently, Black et al. conducted a comprehensive study on 22 adults with a clinical diagnosis of WS, primarily seeking treatment for vestibular complaints [21]. Their extensive evaluation included electronystagmography, rotation testing, visual–vestibular interaction tests, and computerized dynamic posturography. They found that 77% of the WS subjects with vestibular complains had abnormalities in VOR function, vestibulospinal function, or both, highlighting the prevalence of vestibular dysfunction in WS patients. Additionally, Hildesheimer et al. studied 12 adult subjects with WS type 2, recording spontaneous nystagmus and per- and post-rotatory nystagmus [22]. Vestibular hyporeflexia was observed in one subject, minor anomalies in three others, and slight spontaneous nystagmus in seven others, further corroborating the presence of vestibular impairments in WS patients.

Our study represents the largest published cohort of pediatric WS patients to date, all of whom received a precise molecular diagnosis and underwent comprehensive vestibular assessment, including evaluation of otolithic function and inner ear imaging. This combined approach provides, for the first time, a detailed characterization of vestibular involvement in this population, offering important new insights into the underlying genotype–phenotype correlations. Our findings reveal that vestibular dysfunction is highly prevalent in pediatric WS with SNHL, affecting 64% of our cohort. The observed deficit mostly affected the canal sensory organ, with 89% of those with vestibular deficit exhibiting canal dysfunction and 33% showing otolithic dysfunction. These findings emphasize the prevalence of vestibular impairments in WS patients with SNHL early in life. Furthermore, our findings reveal a higher risk of vestibular dysfunction in patients bearing *SOX10* pathogenic variations compared to those without this specific genetic alteration, possibly linked with the inner ear malformation observed in these patients. This observation is noteworthy, as it underscores the critical role of *SOX10* in neurodevelopmental processes, particularly in the context of the inner ear structures. However, it is important to note that these findings are based on small subgroup sizes and multiple comparisons were not corrected for. Nevertheless, these preliminary observations underscore the importance of genetic testing in predicting vestibular outcomes in patients with WS. Further studies with larger cohorts are needed to confirm these associations. Additionally, it is worth noting that one patient with a *MITF* pathogenic variant exhibited complete vestibular loss (areflexia), demonstrating that severe vestibular involvement is not exclusive to WS cases with *SOX10* pathogenic variants.

Age is a well-recognized factor that can influence vestibular function, whether through disease progression, or age-related degeneration. In our cohort, we did not find any significant association between the age at which vestibular testing was performed and the presence of vestibular dysfunction. This lack of association may be explained in part by the fact that our study population was predominantly very young, with a median age of 1.9 years. As such, we cannot exclude the possibility that vestibular function may deteriorate or change over time in patients with WS. While data on the longitudinal evolution of vestibular function in WS are lacking, previous publications have reported cases of progressive HL in WS patients [5]. By analogy, this raises the possibility that vestibular impairment may also follow a progressive course in some individuals with WS. Interestingly, some of the studies examining vestibular dysfunction in adults with WS, including those discussed above, have reported a higher prevalence of vestibular impairment than we observed in our pediatric cohort. Therefore, long-term, longitudinal studies are needed to better understand the natural history of vestibular function in this population.

Although potential confounding factors for vestibular dysfunction, such as comorbid medical conditions, ototoxic medication exposure, trauma, or familial predisposition, could not be comprehensively assessed due to the retrospective design of the study, their impact is likely limited in our cohort. All children included had a genetically confirmed diagnosis of WS, all cases of HL were congenital, and the majority of patients were evaluated early in life. These criteria reduce the likelihood that alternative etiologies or late-onset/acquired causes substantially contributed to the vestibular deficits observed in our population.

A noteworthy proportion of WS patients in our cohort (36%) exhibited normal vestibular function despite their HL. Several factors may explain this, including genotype heterogeneity, variable penetrance, and potential limitations in the sensitivity of current vestibular testing. It is important to note that, when compared to the broader population of children undergoing cochlear implantation for all etiologies, in whom approximately 50% demonstrate normal vestibular function (with a similar level of HL severity) [23], the proportion of normal vestibular function in our WS cohort was noticeably lower. These data suggest that WS children with SNHL may indeed have a higher risk of vestibular impairment compared to the general population of hearing-impaired children, supporting the need for tailored vestibular assessment and counseling within this specific group.

Hearing loss is well-documented in WS. According to a systematic review by Song J et al., HL occurs in 71% of all WS cases: 52.3% in WS type 1, 91.6% in WS type 2, 57.1% in WS type 3, and 83.5% in WS type 4. Pathogenic variations in *SOX10* (96.5%) and *MITF* (89.6%) are more frequently linked to hearing impairment than other WS associated genetic pathogenic variations [5]. In our study, all participants had congenital HL, with a majority of severe to profound bilateral HL. This is likely because vestibular evaluation is routinely performed during HL and pre-cochlear implant evaluations, which may have led to an inclusion bias. As a result, the prevalence of vestibular deficits observed in our study may overestimate the true prevalence in the overall WS population. Moreover, we are unable to provide insights into vestibular function in WS patients with normal hearing or to establish a definite relationship between cochlear and vestibular function. Notably, one patient with unilateral HL exhibited slight vestibular dysfunction in the normal hearing ear, suggesting that the severity of HL may not always indicate the extent of vestibular impairment, and that vestibular dysfunction could even occur in patients with normal hearing.

Temporal bone imaging studies of WS patients have primarily focused on those with WS type 1, WS type 2, or *SOX10* pathogenic variations [2,5,17,18]. While some studies have reported no inner ear malformations in WS type 1 patients [17] others have described inner ear abnormalities in some WS type 1 and WS type 2 patients [18]. However, most inner ear malformations are observed in association with *SOX10* pathogenic variations [2,18]. Reported abnormalities in WS type 1 and WS type 2 patients include enlargement of the vestibular aqueduct, widening of the upper vestibule, narrowing of the internal auditory canal, and decreased modiolus size [18]. The most frequently reported inner ear aberrations associated with *SOX10* pathogenic variations include agenesis or hypoplasia of the SCC, an enlarged or malformed vestibule, and a cochlea with reduced size and occasionally abnormal shape [2]. Additionally, cochlear nerve aplasia can be found in a few patients. Beyond inner ear abnormalities, imaging studies have revealed other associated anomalies such as agenesis of the olfactory bulbs, hypoplastic parotid and lacrimal glands, and white matter signal abnormalities [2]. In our population, we identified inner ear malformation in 55% of cases, with two patients having a *PAX3* pathogenic variation and the remainder being *SOX10* WS2/4 patients. As previously reported, anomaly of the semi-circular canal was the most frequently reported inner ear anomaly. Our data emphasize the association between the presence of inner malformation and the presence of vestibular dysfunction. Even if abnormal vestibular function could be observed in subjects with normal inner ear structures, the prevalence of abnormal vestibular function was clearly increased for those with inner ear malformation.

The *SOX10* gene plays a fundamental role in the development and function of the inner ear, which may explain the increased prevalence and severity of the vestibular deficits observed in WS patients with *SOX10* pathogenic variations compared to those with pathogenic variations in other WS-related genes. SOX10 is a member of the SOX family of transcription factors, which are key regulators of various developmental processes, including sex determination, skeletogenesis, neurogenesis, and neural crest (NC) development [24]. Specifically, the SOX10 transcription factor is a characteristic marker for migratory multipotent NC progenitors, as well as for various NC derivatives. The neural crest is a unique cell population in vertebrates that originates at the neural plate border and migrates along defined pathways to contribute to a wide array of tissues. These include the neurons and glia of the peripheral nervous system, among others. *Sox10* is extensively expressed in the early stages of inner ear development, influencing both cochlear and vestibular structures. It is initially present in the placode-derived otic vesicle, later localizing to the developing epithelium of the cochlea and vestibule, and ultimately becomes restricted to the supporting cells of the neurosensory epithelium [25]. This expression pattern suggests a role for *Sox10* in critical developmental processes such as the survival and differentiation of cochlear progenitors and the proper formation of glial cells within the cochleovestibular ganglia [26]. Transcriptomic analyses have also demonstrated dysregulation of genes involved in cell fate and patterning during inner ear development in the context of SOX10 deficiency [27]. When mutated, *Sox10*/*SOX10* can lead to significant structural abnormalities within the inner ear, which differ depending on the animal model [25,27,28]. These structural changes are consistent with the malformations observed in imaging studies of patients with *SOX10* pathogenic variations, such as agenesis of the vestibulo-cochlear nerve and cochleo-vestibular deformities.

The profound impact of *SOX10* mutations, resulting in inner ear malformation, SNHL, and vestibular deficits, underscores the gene’s essential role in the complex orchestration of inner ear development. By comparison, other genes associated with WS, while crucial for pigmentation and neural crest development, do not have as direct an impact on inner ear structures as *SOX10*. Understanding the unique role of *SOX10* can provide clear insights into targeted diagnostic and therapeutic strategies, emphasizing the need for comprehensive imaging, vestibular, and auditory assessments in affected individuals.

Our regression analysis revealed that only the presence of a SOX10 pathogenic variant, and not vestibular dysfunction, was significantly associated with delayed independent walking in our cohort. Interestingly, contrary to what has been reported in the literature, we did not find a significant independent association between vestibular dysfunction and walking age in our cohort. This result is surprising, as vestibular areflexia is typically considered a strong determinant of delayed acquisition of walking, and all children with vestibular areflexia in previous studies have shown motor delays [23,29]. In our analysis, however, some patients with vestibular areflexia achieved walking at a normal age. Several explanations might be considered: first, it is possible that vestibular dysfunction in some children with WS is progressive rather than congenital, with motor milestones achieved before the full development of vestibular symptoms. Second, unmeasured factors such as compensation by other sensory systems, individual variability in motor development, or additional neurological or environmental influences may play a role, and these factors were not captured in our model. Overall, these findings underscore both the complexity and heterogeneity of motor development in WS, especially in relation to vestibular and genetic status. Limitations remain—particularly the small sample size and potential unmeasured confounding variables—and larger, prospective studies with longitudinal vestibular assessments will be essential to clarify these relationships.

## 5. Conclusions

In this study, we have explored the phenotypic profiles of patients diagnosed with WS, with a particular focus on vestibular function. Our data reveal a significant prevalence of vestibular deficits in WS patients with SNHL, even at early ages, highlighting a critical yet often overlooked aspect of the syndrome. The profound influence vestibular dysfunction can have on motor and cognitive development underscores the urgent need for comprehensive vestibular assessments as part of routine clinical evaluations for all WS patients.

Furthermore, our data demonstrate a higher prevalence and severity of vestibular deficits in WS patients with *SOX10* pathogenic variations compared to those with mutations in other WS-related genes. This finding is consistent with the unique and pivotal role of *SOX10* in inner ear development and function, making it a crucial target for diagnostic and therapeutic strategies. The link between *SOX10* pathogenic variations and vestibular dysfunction underscores the importance of molecular analysis in confirming the clinical diagnosis of WS and informing patients and clinicians about potential risks of vestibular deficits. While *SOX10* pathogenic variations are typically linked to vestibular dysfunction, it should be emphasized that severe vestibular involvement, with significant clinical and developmental repercussions, may also be found with other WS-related genes, including *MITF*.

In conclusion, our research highlights the essential role of comprehensive vestibular evaluations in the management of WS with SNHL. Incorporating routine vestibular evaluations into clinical care can enable earlier detection and targeted interventions, ultimately improving quality of life for WS patients. Furthermore, extending vestibular assessment to WS patients with normal hearing is crucial for determining whether vestibular dysfunction occurs independently of HL. This approach will not only improve patient outcomes, but also advance our understanding of the intricate relationship between genetic mutations and inner ear function in WS.

## Figures and Tables

**Table 1:** Genetic, vestibular, audiometric, and radiological findings in our patients with WS.

Subject			WS		Canal Function						Otolithic Function			Hearing		Imaging	
Subject	Sex	Age (Years)	Mutated Gene	WS Type	HIT	vHIT	Rotatory Chair (Manual)	Rotatory Chair (Recorded)	Caloric Test	Global	cVEMP Right	cVEMP LEFT	Global	Deafness Side	HL	Cochlear Malformation	Vestibular/ SCC Malformation
1	F	1.0	*EDNRB*	2	N	P	N	*	N	P	PRESENT	PRESENT	N	B	PROFOUND	No	No
2	M	1.9	*EDNRB*	4	*	*	*	*	N	N	*	*	*	B	PROFOUND	No	No
3	F	0.8	*MITF*	2	N	*	N	N	N	N	PRESENT	PRESENT	N	B	SEVERE	No	No
4	F	1.3	*MITF*	2	N	*	N	N	N	N	PRESENT	PRESENT	N	B	PROFOUND	No	No
5	F	0.5	*MITF*	2	N	N	N	*	N	N	PRESENT	PRESENT	N	B	PROFOUND	No	No
6	F	11.6	*MITF*	2	A	*	A	A	A	A	ABSENT	ABSENT	A	B	PROFOUND	No	No
7	M	0.8	*PAX3*	1	N	*	*	*	N	N	PRESENT	PRESENT	N	B	PROFOUND	No	No
8	M	2.5	*PAX3*	1	N	N	N	*	P	P	PRESENT	PRESENT	N	B	PROFOUND	No	No
9	M	1.9	*PAX3*	1	N	*	N	N	N	N	PRESENT	PRESENT	N	B	SEVERE	No	Yes
10	F	3.3	*PAX3*	1	P	*	N	*	N	N	PRESENT	PRESENT	P	B	PROFOUND	No	No
11	M	12.2	*PAX3*	1	N	*	N	N	N	N	PRESENT	PRESENT	N	B	SEVERE	No	No
12	M	9.7	*PAX3*	1	N	*	N	N	P	P	*	*	*	B	PROFOUND	No	Yes
13	F	3.4	*PAX3*	1	N	*	N	*	N	N	PRESENT	PRESENT	N	B	PROFOUND	No	No
14	M	0.6	*PAX3*	1	N	*	N	N	P	P	PRESENT	PRESENT	N	B	PROFOUND	No	No
15	M	1.5	*PAX3*	1	N	*	*	*	N	N	PRESENT	PRESENT	N	U	N+PROFOUND	No	No
16	M	10.9	*PAX3*	1	*	N	N	*	N	N	*	*	P	U	N+PROFOUND	No	No
17	M	1.9	*PAX3*	1	N	*	*	*	N	N	*	*	*	B	PROFOUND	No	No
18	M	1.8	*SOX10*	2	N	*	*	*	P	P	PRESENT	PRESENT	N	B	PROFOUND	Yes	Yes
19	M	0.7	*SOX10*	2	P	P	N	*	N	P	PRESENT	PRESENT	N	B	PROFOUND	Yes	Yes
20	F	2.6	*SOX10*	2	A	*	A	*	*	A	PRESENT	PRESENT	N	B	PROFOUND	Yes	Yes
21	M	0.9	*SOX10*	2	A	P	A	*	A	P	PRESENT	PRESENT	N	B	PROFOUND	Yes	Yes
22	M	0.3	*SOX10*	2	N	N	N	*	N	N	PRESENT	PRESENT	N	B	PROFOUND	Yes	Yes
23	F	1.9	*SOX10*	2	A	*	N	*	P	P	PRESENT	PRESENT	N	B	PROFOUND	Yes	Yes
24	M	3.2	*SOX10*	4	A	*	A	A	A	A	ABSENT	ABSENT	A	B	PROFOUND	Yes	Yes
25	M	5.2	*SOX10*	4	A	*	A	A	*	A	*	*	*	B	PROFOUND	Yes	Yes
26	M	2.7	*SOX10*	4	N	*	N	P	P	P	PRESENT	PRESENT	N	B	PROFOUND	Yes	Yes
27	M	0.9	*SOX10*	2	P	*	*	*	P	P	PRESENT	PRESENT	N	B	PROFOUND	Yes	Yes
28	M	1.8	*SOX10*	2	P	*	P	A	A	P	PRESENT	PRESENT	N	B	PROFOUND	Yes	Yes
29	M	5.6	*SOX10*	2	P	*	N	N	N	P	PRESENT	PRESENT	P	B	PROFOUND	Yes	Yes
30	M	8.6	*SOX10*	2	P	*	A	A	P	P	PRESENT	PRESENT	N	B	PROFOUND	Yes	Yes
31	M	6.5	*SOX10*	2	A	*	A	A	A	A	*	*	*	B	PROFOUND	Yes	Yes
32	M	9.4	*SOX10*	4	N	*	*	*	N	N	PRESENT	PRESENT	N	U	N+MILD	Yes	Yes
33	M	1.7	*SOX10*	2	P	*	*	N	N	P	*	*	*	B	PROFOUND	No	Yes
34	F	1.5	*SOX10*	2	N	P	P	*	P	P	PRESENT	ABSENT	P	B	PROFOUND	No	Yes

N: Normal; P: Partial; A: Areflexia/Total Absence of function; B: Bilateral; U: Unilateral; *: Test not performed; HIT: Head Impulse Test; vHIT: video Head Impulse Test; cVEMP: cervical Vestibular Myogenic Potential; HL: Hearing Loss.

**Table 2:** Distribution of the WS patients included in this study over genes and specific WS phenotypes. Results are expressed in number of subjects.

	WS Type	Type 1	Type 2	Type 4	Total
Gene					
*PAX3*		11			**11**
*MITF*			4		**4**
*SOX10*			13	4	**17**
*EDNRB*			1	1	**2**

## Data Availability

Data can be made available by the authors upon reasonable request.

## Abbreviations

The following abbreviations are used in this manuscript:

ABR	Auditory Brainstem Response
ASSR	Auditory Steady-state Response
BIAP	International Bureau of Audiophonology
cVEMP	cervical Vestibular Evoked Myogenic Potential
FLAIR	Fluid Attenuated Inversion Recovery
HIT	Head Impulse Test
HL	Hearing Loss
NC	Neural Crest
PCWH	Peripheral demyelinating neuropathy
SCC	Semicircular Canal
SNHL	Sensorineural Hearing Loss
TEOAE	Transient Evoked Otoacoustic Emissions
VOR	Vestibulo-Ocular Reflex
vHIT	video Head Impulse Test
WS	Waardenburg Syndrome

## References

[1] Waardenburg P.J. (1951). A new syndrome combining developmental anomalies of the eyelids, eyebrows and nose root with pigmentary defects of the iris and head hair and with congenital deafness. Am. J. Hum. Genet..

[2] Elmaleh-Bergès M., Baumann C., Noël-Pétroff N., Sekkal A., Couloigner V., Devriendt K., Wilson M., Marlin S., Sebag G., Pingault V. (2013). Spectrum of temporal bone abnormalities in patients with Waardenburg syndrome and SOX10 mutations. AJNR Am. J. Neuroradiol..

[3] Pingault V., Ente D., Dastot-Le Moal F., Goossens M., Marlin S., Bondurand N. (2010). Review and update of mutations causing Waardenburg syndrome. Hum. Mutat..

[4] Maudoux A., Vitry S., El-Amraoui A. (2022). Vestibular Deficits in Deafness: Clinical Presentation, Animal Modeling, and Treatment Solutions. Front. Neurol..

[5] Song J., Feng Y., Acke F.R., Coucke P., Vleminckx K., Dhooge I.J. (2016). Hearing loss in Waardenburg syndrome: A systematic review. Clin. Genet..

[6] Wiener-Vacher S.R., Hamilton D.A., Wiener S.I. (2013). Vestibular activity and cognitive development in children: Perspectives. Front. Integr. Neurosci..

[7] Doubaj Y., Pingault V., Elalaoui S.C., Ratbi I., Azouz M., Zerhouni H., Ettayebi F., Sefiani A. (2015). A novel mutation in the endothelin B receptor gene in a Moroccan family with Shah-Waardenburg syndrome. Mol. Syndromol..

[8] Léger S., Balguerie X., Goldenberg A., Drouin-Garraud V., Cabot A., Amstutz-Montadert I., Young P., Joly P., Bodereau V., Holder-Espinasse M. (2012). Novel and recurrent non-truncating mutations of the MITF basic domain: Genotypic and phenotypic variations in Waardenburg and Tietz syndromes. Eur. J. Hum. Genet..

[9] Mousty E., Issa S., Grosjean F., Col J.Y., Khau Van Kien P., Perez M.J., Petrov Y., Reboul D., Faubert E., Le Gac M.-P. (2015). A homozygous PAX3 mutation leading to severe presentation of Waardenburg syndrome with a prenatal diagnosis. Prenat. Diagn..

[10] Bondurand N., Dastot-Le Moal F., Stanchina L., Collot N., Baral V., Marlin S., Attie-Bitach T., Giurgea I., Skopinski L., Reardon W. (2007). Deletions at the *SOX10* gene locus cause Waardenburg syndrome types 2 and 4. Am. J. Hum. Genet..

[11] Bertani-Torres W., Lezirovitz K., Alencar-Coutinho D., Pardono E., da Costa S.S., Antunes L.d.N., de Oliveira J., Otto P.A., Pingault V., Mingroni-Netto R.C. (2023). Waardenburg Syndrome: The Contribution of Next-Generation Sequencing to the Identification of Novel Causative Variants. Audiol. Res..

[12] PFMG2025 Contributors (2025). PFMG2025-integrating genomic medicine into the national healthcare system in France. Lancet Reg. Health Eur..

[13] MLPA: Multiplex Ligation-Dependent Probe Amplification MRC Holland. https://www.mrcholland.com/technology/mlpa.

[14] Pingault V., Bodereau V., Baral V., Marcos S., Watanabe Y., Chaoui A., Fouveaut C., Leroy C., Vérier-Mine O., Francannet C. (2013). Loss-of-function mutations in *SOX10* cause Kallmann syndrome with deafness. Am. J. Hum. Genet..

[15] Jongkees L.B., Maas J.P., Philipszoon A.J. (1962). Clinical nystagmography. A detailed study of electro-nystagmography in 341 patients with vertigo. Pract. Otorhinolaryngol..

[16] Chan F.M., Galatioto J., Amato M., Kim A.H. (2016). Normative data for rotational chair stratified by age. Laryngoscope.

[17] Oysu C., Oysu A., Aslan I., Tinaz M. (2001). Temporal bone imaging findings in Waardenburg’s syndrome. Int. J. Pediatr. Otorhinolaryngol..

[18] Madden C., Halsted M.J., Hopkin R.J., Choo D.I., Benton C., Greinwald J.H. (2010). Temporal bone abnormalities associated with hearing loss in Waardenburg syndrome. Laryngoscope.

[19] Marcus R. (1968). Vestibular function and additional findings in Waardenburg’s syndrome. Acta Otolaryngol..

[20] Hageman M.J., Oosterveld W.J. (1977). Vestibular Findings in 25 Patients With Waardenburg’s Syndrome. Arch. Otolaryngol..

[21] Black F.O., Pesznecker S.C., Allen K., Gianna C. (2001). A vestibular phenotype for Waardenburg syndrome?. Otol. Neurotol..

[22] Hildesheimer M., Maayan Z., Muchnik C., Rubinstein M., Goodman R.M. (1989). Auditory and vestibular findings in Waardenburg’s type II syndrome. J. Laryngol. Otol..

[23] Jacot E., Van Den Abbeele T., Debre H.R., Wiener-Vacher S.R. (2009). Vestibular impairments pre- and post-cochlear implant in children. Int. J. Pediatr. Otorhinolaryngol..

[24] Pingault V., Zerad L., Bertani-Torres W., Bondurand N. (2022). SOX10: 20 Years of phenotypic plurality and current understanding of its developmental function. J. Med. Genet..

[25] Breuskin I., Bodson M., Thelen N., Thiry M., Borgs L., Nguyen L., Lefebvre P.P., Malgrange B. (2009). Sox10 promotes the survival of cochlear progenitors during the establishment of the organ of Corti. Dev. Biol..

[26] Breuskin I., Bodson M., Thelen N., Thiry M., Borgs L., Nguyen L., Stolt C., Wegner M., Lefebvre P.P., Malgrange B. (2010). Glial but not neuronal development in the cochleo-vestibular ganglion requires Sox10. J. Neurochem..

[27] Hao Q.Q., Li L., Chen W., Jiang Q.Q., Ji F., Sun W., Wei H., Guo W.-W., Yang S.-M. (2018). Key Genes and Pathways Associated With Inner Ear Malformation in *SOX10* p.R109W Mutation Pigs. Front. Mol. Neurosci..

[28] Dutton K., Abbas L., Spencer J., Brannon C., Mowbray C., Nikaido M., Kelsh R.N., Whitfield T.T. (2009). A zebrafish model for Waardenburg syndrome type IV reveals diverse roles for Sox10 in the otic vesicle. Dis. Model. Mech..

[29] Wiener-Vacher S.R., Campi M., Caldani S., Thai-Van H. (2024). Vestibular Impairment and Postural Development in Children with Bilateral Profound Hearing Loss. JAMA Netw. Open.

